# Decreased postural control in people with moderate hearing loss

**DOI:** 10.1097/MD.0000000000010244

**Published:** 2018-04-06

**Authors:** Ewan Thomas, Francesco Martines, Antonino Bianco, Giuseppe Messina, Valerio Giustino, Daniele Zangla, Angelo Iovane, Antonio Palma

**Affiliations:** a Sport and Exercise Sciences Research Unit; b Bio.Ne.C. Department, ENT Section, University of Palermo; c PosturaLab Center, Palermo, Italy.

**Keywords:** EMG, falls, hearing loss, posture, range of movement

## Abstract

Balance is a complex process that involves multiple sensory integrations. The auditory, visual, and vestibular systems are the main contributors. Hearing loss or hearing impairment may induce inappropriate postural strategies that could affect balance and therefore increase the risk of falling.

The aim of this study was to understand whether hearing loss could influence balance, cervical posture, and muscle activation in the cervical region.

Thirteen patients (61 ± 13 years; 161.8 ± 11.0 cm; 70.5 ± 15.9 kg) with moderate hearing loss (Right ear −60 ± 21 dB; Left ear −61 ± 24 dB) underwent: an audiometric examination, a postural examination (with open and closed eyes) through a stabilometric platform, a cervical ROM examination through a head accelerometer, and a sternocleidomastoid electromyography (EMG) examination.

A linear regression analysis has shown a regression coefficient (*R*^2^) 0.76 and 0.69 between hearing loss and the posturographic parameters, on the sagittal sway, with open and closed eyes, respectively. The combination of frontal and sagittal sway is able to explain up to 84% of the variance of the audiometric assessment. No differences were found between right and left hemibody between the audiometric, posturographic, cervical ROM parameters, and in EMG amplitude. ROM and EMG parameters have not shown any significant associations with hearing loss, for both right and left head rotation.

Hearing loss is associated to increased posturographic measures, especially the sagittal sway, underlining a reduced postural control in people with hearing impairments. No association was found between the heads posture and neck activation with hearing loss. Hearing loss may be associated with an increased risk of falls.

## 1 Introduction

Falls are a major risk of accidental death for people over 65 years of age. It has been well established by various epidemiological studies that hearing loss may be a contributor for the increased number of falls in the elderly population. The National Health and Nutrition Examination Survey (2001–2004) database has shown that for each 10 dB of hearing loss, individuals have a 1.4 time increased risk of falling.^[1,2]^

Falling is of course conditioned by the ability of each individual to properly balance and overcome transient unbalances. The ability of the body to balance is related to its center of mass and the area of the base in which the body is balancing. If the line of gravity falls in the base of the object, then that object will be in balance. If the line of gravity falls out of the base of the object, then it will result in an imbalance and in case of a person, this will fall. In order to increase the stability of an object it will be necessary to act on the base of the object or on its center of gravity.^[3]^ In a human being it is not possible to act on its base; thus, the only way to ensure proper balance is to act on its center of gravity through an adequate postural control. The ability of the body to balance is aided by various sensory systems, which find the proprioceptive, vestibular, visual, and auditory systems as main contributors.^[4]^ All the sensory systems, including hearing acuity, reduce their capacity throughout aging and such functional decrease is also related with an increased risk of falls.^[2,5–7]^ Postural sway, considered like spontaneous shifts during a standing position, represents the output of integration of the sensory systems that contribute to the body's posture.^[8]^ Other reasons by which elderly people may lose balance, besides the loss of sensory inputs, may be inappropriate gate, cognitive impairments,^[9]^ or different comorbidities arising from several pathologies.^[10]^

Balance is also influenced by sound stimuli. Such influence can be considered either positive or negative, depending on the intensity and frequency of the sound stimulus. In the study of Alessandrini et al,^[11]^ a sound stimulus of 500 Hz at 130 dB was delivered monoaureally to normal subjects, through telescopic headphones, during a postural sway analysis. The authors then compared the outcomes to a no-sound condition. Their results showed differences during the sound and the sound-free task on the postural sway expressed on the frontal plane, in relation to the direction which the sound stimulus was applied, resulting in an increase of the body sway. Same research experiment was carried out by Mainenti et al using 2 different frequencies of 500 and 4000 Hz at 70 dB. No differences were however observed in these conditions between the sound and the silent task. Further evidence about the influence of sound frequency on postural control arises from the study of Siedlecka et al, in which the authors conclude that low frequencies do not have any influence on balance, but sounds delivered between 1000 and 4000 Hz at 80 dB are able to improve body sway on the anterior-posterior plane in healthy subjects. Thus, loud sounds have been seen to negatively influence balance, whereas sounds ranging between 1000 and 4000 Hz may act improving subjects stability.^[11–13]^ However, such findings have been carried out on healthy subjects and therefore it is not possible to generalize such conclusions in people with hearing impairments. Although, when direct comparisons are made between subjects with normal and impaired hearing, these latter seem to have a lower postural control regardless of age and sex.^[10,14,15]^ In addition, balance may also be influenced by the position of the head in respect to the body and an altered head position has been seen as a frequent condition in patients suffering of sensineural hearing loss.^[8,16]^ There is a close relation between the proprioceptive receptors located in the neck and the vestibular system and these together determine the sense of head position and hence the alignment expressed by the body's posture.^[8,17]^ Such strict relation may also explain why those who suffer cervical dystonia manifest postural instability, affecting static, dynamic balance, and gait and refer fear of falling.^[18]^ It is therefore, the main aim of this work to understand the relationship between hearing loss and static balance and secondly to understand whether there are further relations between hearing loss and the head posture and the muscular activation of the neck.

## 2 Methods

### 2.1 Participants

Eighteen participants were initially retained for investigation (56 ± 18 years; 162.7 ± 11.9 cm; 69.7 ± 15.5 kg). However, 5 had to be excluded for issues related to traumas and balance disorders. Thirteen participants (61 ± 13 year; 161.8 ± 11.0 cm; 70.5 ± 15.9 kg) with hearing loss (right ear −60 ± 21 dB pure tone average [PTA]; left ear −61 ± 24 dB PTA) were ultimately retained for investigation. The participants were selected in the ENT unit of the University's Hospital.

A cross-sectional experimental study was adopted to investigate the influence of hearing loss on postural parameters, neck rotation, and sternocleidomastoid activation. Hearing loss was considered the independent variable.

Before data collection, written informed consent was obtained from all subjects, and the university's institutional review board for the protection of human subjects approved the investigation.

Individuals with a history of balance disorders or complains of imbalance and/or clinical signs related to vestibular disorders, individuals with traumas or individuals taking medications that could alter postural parameters or the central nervous system, or individuals with chronic muscular-skeletal pathologies were excluded from investigation. Additionally, individuals with recent surgical history were excluded from investigation. Participants with sudden hearing loss were also excluded from investigation. The study was performed in compliance with the Helsinki Declaration.

### 2.2 Data collection

The participants underwent: a hearing examination to address the presence of hearing impairments or diseases of the ear and subsequently, posture through a baropodometric platform to evaluate balance and postural control, neck rotation to address the range of movement (ROM) of the neck, and a sternocleidomastoid electromyographic assessment (EMG) to evaluate the amplitude of muscular activation. The tests were administered in the above listed order. All tests were administered between 9 and 12 am. All hearing examinations were administered by the same physician of the ENT unit, and all the other tests by another skilled investigator.

### 2.3 Audiometric assessment

Pure-tone air- and bone-conduction average thresholds were measured for the frequencies 0.25–0.5–1–2–4–8 kHz for both left ear and right ear and the air measures were used to classify hearing loss degree as follows: normal hearing (<20 dB); mild hearing loss (21–40 dB); moderate hearing loss (41–70 dB); severe hearing loss (71–90 dB); profound hearing loss (>90 dB).^[19]^ The assessment was undertaken in a sound-isolated booth. All patients were free of otologic diseases.

### 2.4 Postural assessment

For the posturographic assessment, each participant performed the sway test using standardized Romberg positioning: the feet were placed side-by-side, forming an angle of 30 degree and both heels were 4 cm apart. Posturography was measured using the FreeMed posturography system, including the FreeMed baropodometric platform and the FreeStep v.1.0.3 software. The system was set to sample postural sway at 100 Hz. The sensors, coated with 24K gold, guarantee repeatability and reliability of the instrument (produced by Sensor Medica, Guidonia Montecelio, Roma, Italy). Participants were asked to perform the standardized Sway test on the baropodometric platform. The assessment was undertaken in the same sound-isolated booth of the audiometric assessment. Data from the platform were converted in accordance with instructions provided by the manufacturer and transformed into coordinates of the center of pressure (CoP). Participants repeated the static standing measures with eyes open (OE) during the first analysis and with closed eyes (CE) during the second analysis. The following parameters of the statokinesigram were considered for both OE and CE: length of the sway of the CoP; ellipse surface area (SE); these derive from the coordinates of the CoP along the frontal (X; right-left; X-mean) and sagittal (Y; forward-backward; Y-mean) planes.^[20]^

### 2.5 Cervical ROM evaluation

Cervical ROM was evaluated via a noninvasive technique using an accelerometer (mOOver, Sensor Medica; Guidonia Montecelio, Roma, Italia), a wireless electronic, computer-aided measuring device using the FreeStep software (Sensor Medica; Guidonia Montecelio, Roma, Italia). The mOOver accelerometer allows measurements of range of motion, acceleration values, and total amount of motion on the X, Y, and Z planes. Each individual was seated in a standardized chair (length: 40 cm; breadth: 40 cm; height: 45 cm) with their back at a 90-degree angle, with the sacrum and the shoulder blades adhering to the backrest, feet flat on the floor, hands on the thighs, and the head in a neutral position. To assess cervical ROM, the device was positioned medially on the head, at the level of the frontal bone, above the bridge of the nose, and then fastened to the head through a strap. Verbal commands were given to the participants to perform neck movements until the maximal ROM was achieved. The participants were asked to rotate the head to the left and to the right at the maximal possible rotation. Such measurement was performed during the maximal voluntary contraction (MVC) of the EMG evaluation, to evaluate neck rotation and sternocleidomastoid activation during the same task. All assessments were performed 3 times.

Subsequently the participants were asked to rotate the head at their preferred rotational degree, to mimic ordinary life tasks, and maintain such rotation for 30 seconds, both on the right and left side. EMG recordings were assessed at the same time.

The maximum rotation was retained for investigation for both the right and left rotation. Average measures were considered for the 30-second tasks and a percentage value according to the maximal rotation was used for data comparison.

### 2.6 Electromyographic evaluation

#### 2.6.1 Maximal voluntary contraction

The maximal force exerted by the sternocleidomastoid muscle was determined by asking the participants to increase the force from rest to maximum gradually in ∼3 seconds and to then maintain the maximum for an additional 3 seconds. Repeated contractions were performed until 2 attempts were within 5% of each other and the greater peak force was used as the subject's MVC force.

#### 2.6.2 EMG

Sternocleidomastoid activation was assessed through surface EMG (Quattro, OTBioelettronica, 2010, IT) through the OTBioLab software. Surface bipolar electrodes were applied on the sternocleidomastoid muscles far away from the innervations zones of each muscle.^[21]^ The participants after the MVC assessments were asked to rotate their head on both the left and right side of their body at their preferred head rotation for 30 seconds. Recordings were made during the rotational tasks. The recorded signal was filtered through a band pass filter between 20 and 400 Hz, the output signal was subsequently rectified and the root mean square was taken into account for amplitude analysis. Mean bilateral muscle activity was taken into account for each muscle group of both sides. EMG data are expressed as the root mean square and then normalized to the EMG obtained during the MVC.

### 2.7 Statistical analysis

Statistical analysis was performed using Student *t* test for paired data and the Wilcoxon test for nonparametric assessments. Pearson correlation test was used when appropriate. A regression analysis was performed to determine the predictor variables associated to hearing loss. An alpha value <0.05 was considered statistically significant. The STATISTICA software (StatSoft. Tulsa, ver.12) was used for all statistical analysis.

## 3 Results

**Figure 1:**
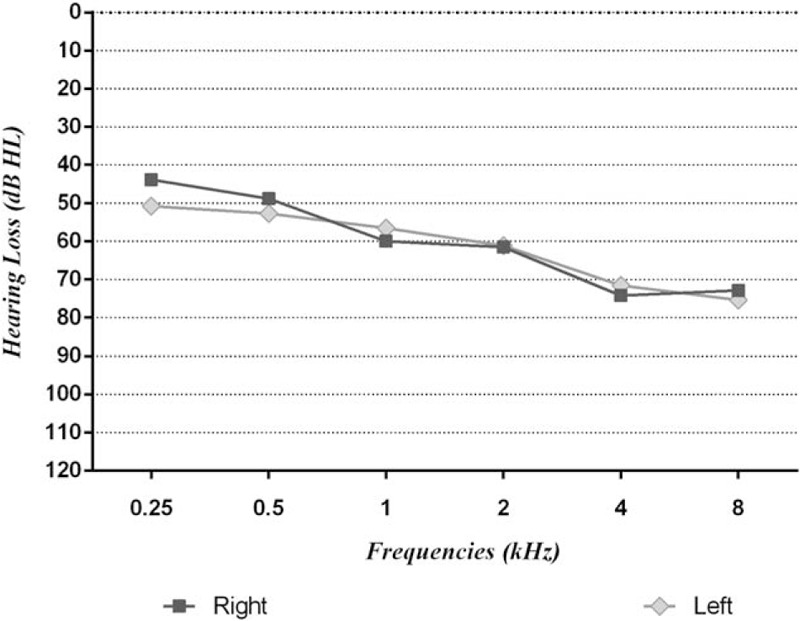
Audiometric parameters of the sample. The hearing loss expressed in dB of the left and right ear are shown for each sampled frequency.

Main descriptive results are summarized in Table 1 and the audiometric outcomes in Figure 1 in which it is possible to note a concomitant reduction of both the frequency range and the sound intensity for both ears. No differences were found between left and right hemibody for neither head rotation (*P* = .56), EMG activation (*P* = .92) and the audiological assessment (*P* = .90). No association was found between head ROM and EMG parameters (*r* = 0.27 between left MVC and right head rotation and −0.18 with right MVC and left head rotation) nor between ROM and the audiological measures (*r* = −0.28 between right head rotation and right PTA measures and *r* = 0.19 between left head rotation and left PTA measures) neither between the EMG and the audiological measures (*r* = 0.38 and 0.05 between right PTA and right MVC and its percentage activation for the 30-second task, respectively and *r* = 0.48 and −0.26 between left PTA and right MVC and its percentage activation for the 30-second task, respectively). No relevant association was found between head ROM and the posturographic parameters (associations between left and right head rotation and the length of the CoP, the SE, and the sway on both the frontal and sagittal plane) and between EMG and posturographic parameters (associations between left and right MVC and the percentage activation during the 30-second task and the length of the Cop, the area of the ellipse, and oscillations on both the frontal and sagittal plane). All the associations with the posturographic measures have moderate to low correlation indexes, with no statistical significance. Right head rotation showed a correlation coefficient of *r* = 0.82 with left head rotation during the 30-second rotation task as a percentage of muscle function.

**Table 1:**
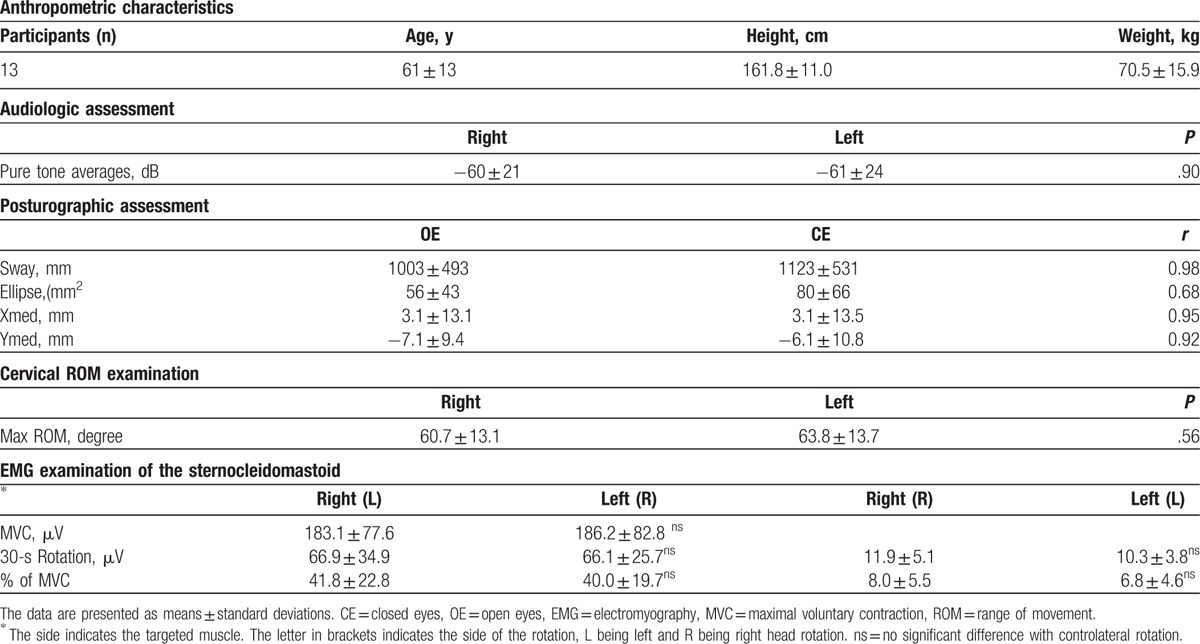
Main descriptive results.

The posturographic parameters showed high correlations between the OE and CE measurements (*r* = 0.95 between OE vs. CE of the frontal plane sway and *r* = 0.94 between OE vs. CE of the sagittal plane sway for the CoP measures, whereas the length of the CoP between OE vs. CE showed a correlation coefficient of *r* = 0.98; the SE measures between OE vs. CE showed a correlation coefficient of *r* = 0.68 with a greater SE during the CE measure).

Significant linear regression coefficients (*R*^2^ = 0.76, *P* < .05 and *R*^2^ = 0.68, *P* < .05) were found between the mean frequency PTA measures of the sample and the sway measures on the sagittal plane, during the OE and CE task, respectively. Other variables of interest that account for a significative amount of variance of the PTA frequency measures are the CoP and SE, together, in both the OE and CE task. Further results are summarized in Table 2.

**Table 2:**
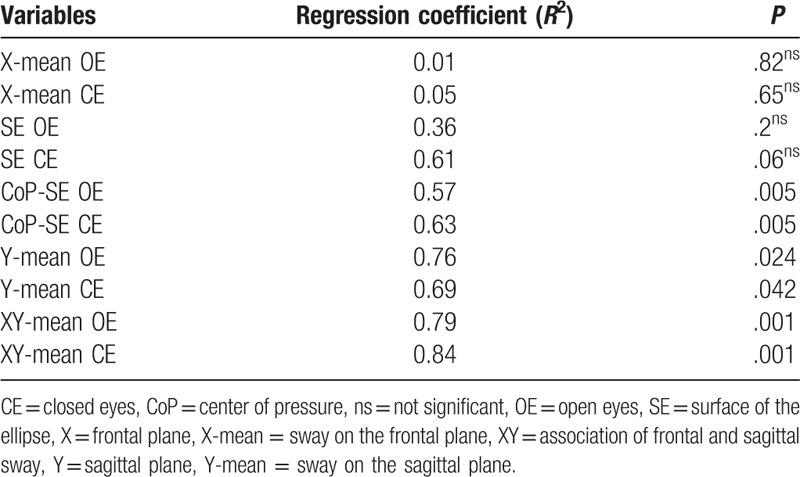
Regression coefficients between the PTA frequency and the postural measures.

## 4 Discussion

The main purpose of this study was to examine the association between hearing loss and balance in patients with moderate hearing loss, and secondly to evaluate a possible associations between cervical ROM and EMG amplitude with postural parameters.

Our main results underline that hearing loss negatively acts on postural control influencing the anterior-posterior sway, whereas it does not seem that hearing loss affects the activation strategies of the neck, neither its range of motion. Our results seem to be in line with those from other studies in regard to the association between hearing loss and balance.^[2,13,22]^ A recent study analyzed postural sway in a sample of people to which a sound stimulus of various levels of intensity and frequency was delivered.^[22]^ In particular, the authors revealed that the length of the sway significantly increased when the frequency of the sound increased, especially on the anterior-posterior axis, but there was not a significative effect in relation to the intensity of the sound stimulus. The frequencies that mainly affected postural control during the experiment were those of 3000 and 4000 Hz. The results carried out by Siedlecka et al^[13]^ support the hypothesis that postural control is mainly affected by sound frequency rather than sound loudness. The authors highlight a decrease of the CoP during the sound stimulus task in particular on the anterior-posterior plane. The cohort of Siedlecka et al was formed by normal subjects whereas our sample by patients with sensorineural hearing loss; notwithstanding the different populations, it is on similar level of frequencies that the anterior-posterior sway seems to be altered during quiet standing.

Balance relies on continuous afferent inputs from multisensory modalities (visual, vestibular, somatosensory, and auditory) and a reduction of any of these leads to a feedback reduction in controlling balance.^[23]^ Peterka^[24]^ in 2002 proposed a model for sensory interaction to maintain balance, proposing a 70% dependency of the central nervous system (CNS) on somatosensory integration, 20% on vestibular integration, and 10% on visual integration. However, it is known that healthy subjects are able to maintain body orientation and motion in space with auditory stimuli alone.^[25]^ Therefore, auditory cues are able to influence postural alignment, and postural alignment alters the ability to locate auditory cues.^[23–25]^ An experiment carried out by Stevens et al^[26]^ has tried to define the contribution of auditory stimuli to maintain balance. The experiment consisted of positioning 2 samples of people, a normal and a balanced impaired population, in an isolated booth and selectively add or remove a sensorial stimulus (Subjects were tested in the dark and in the light, with and without spatial sound cues, and on stable or unstable support surfaces). Stevens et al conclude that sound cues were able to improve the ability of both populations to balance, and that sound alone would lead to an improvement of the subjects body sway to less than two-thirds compared to the no-sound condition, but only during the vision deprived tasks. A similar experiment conducted by Dozza et al^[25]^ has demonstrated that auditory biofeedback was more beneficial in reducing postural sway when visual and somatosensory inputs were reduced, underlining that the degree on which the CNS relies on auditory stimuli to maintain balance depends on the degree of visual, vestibular and somatosensory loss. Sound would seem to work as a spatial landmark that helps the other sensory inputs to recreate a 3-dimensional multisensory environment. It is interesting to note that auditory biofeedback training is used as a neurological tool in people with balance impairments such as hemiplegic patients,^[27]^ mild traumatic brain injury patients,^[28]^ and healthy subjects,^[29]^ to improve balance. In all these diverse conditions, the addition of a sound stimulus leads to a reduction of postural sway. In particular, sound cues, have been seen to elicit responses on the anterior posterior plane in either normal^[30]^ and balance impaired subjects.^[31]^ It is then plausible that in a population subjected to physiological hearing loss, such auditory reduction would lead to an increase of their body sway. Such hypothesis is confirmed by Melo Rde et al^[14]^ who analyzed the posturographic parameters between subjects with normal hearing and subjects with sensorineural hearing loss on stable and unstable surfaces, and concluded that on both surfaces the subjects with sensorineural hearing loss had greater instability compared to their healthy counterparts. Such instability was reported to be concomitant to the increase of the hearing loss.

Russolo^[32]^ investigated the effects of postural sway with different head positions and showed that in a sample of normal subjects an acoustic stimulus, delivered monaurally, of medium-high frequency and high intensity, caused postural responses. Despite each head position caused the body's postural sway to go in different directions, this latter parameter was mainly associated to the side the sound stimulus was applied. The results of the study of Russolo indicate that sounds affect postural control with a greater magnitude compared to heads posture. In line with the research of Russolo our results also seem to underline no association between head rotation and the postural parameters.

Different efforts have been also made to associate posture and muscular activation patterns.^[33,34]^ Functionally, neck muscles can be divided into superficial and deep muscles. Superficial muscles, such as the sternocleidomastoid, that we analyzed in our study, are engaged during wide movements.^[35]^ Deep muscles control neck position during static posture, also avoiding compressive loads.^[35,36]^ However, Jull et al^[37]^ showed that people with neck pain, to compensate the weakness of the deeper muscles, are more likely to overwork their superficial neck muscles. In addition, the study of Mang et al has analyzed the effect of a pre-impact sound at a high intensity presented before a whiplash like perturbation and the results showed that after the loud tone, neck muscles activity decreased. However, such phenomenon was not present in all the analyzed muscles, in particular in the sternocleidomastoid muscle the decreased amplitude was not significant compared to the whiplash like perturbation presented without the sound stimulus. Such result could provide explanation in regards our outcome on the EMG analysis that seems to exclude the association between muscular activation and the posturographic parameters in subjects with hearing loss. It is in light of these considerations that it is not possible to state whether our results are because of a methodological limit, considering that our EMG analysis was extracted from surface electrodes on a relatively superficial muscle, or whether hearing loss may be associated to muscle activation in other neck muscles, such as the cervical paraspinal muscles presented by Wang et al.

There also seems to be a relation between the left and right side of the body in regard to head's rotation. Such relation may also represent a limit for the interpretation of the results, notwithstanding the statistical outcomes seem to confirm no relation between ROM and EMG parameters.

It has to be also noted that our results are based on a sample of 13 participants that represent a confined number of patients to draw general conclusions and that vestibular function has not been directly assessed.^[38]^ Notwithstanding the clinical examination of each patient has been carried out by a ENT physician, clinical signs, and absence of imbalance alone may not objectively exclude a vestibular dysfunction.^[38]^

The results of this study indicate that hearing loss is primarily associated to a greater sagittal sway and secondly to the association between the frontal and sagittal sway. Such association does not change in both open and closed eye conditions. There does not seem to be any influence between posture and head rotational parameters and between posture and EMG parameters. It also seems that hearing loss does not influence the latter parameters.

It is therefore advisable to program proper postural interventions in subjects with hearing loss in order to avoid falls that could increase the risk of mortality especially in an elderly population physiologically subjected to increased hearing loss.

## Author contributions

**Conceptualization:** A. Bianco, A. Palma, E. Thomas.

**Data curation:** F. Martines.

**Formal analysis:** V. Giustino.

**Investigation:** E. Thomas, F. Martines.

**Methodology:** D. Zangla, V. Giustino.

**Resources:** G. Messina.

**Supervision:** A. Bianco, G. Messina.

**Validation:** A. Iovane, A. Palma.

**Writing – original draft:** A. Iovane, E. Thomas.

**Writing – review & editing:** E. Thomas.
